# Data-Driven Computational Social Network Science: Predictive and Inferential Models for Web-Enabled Scientific Discoveries

**DOI:** 10.3389/fdata.2021.591749

**Published:** 2021-04-22

**Authors:** Frank Emmert-Streib, Matthias Dehmer

**Affiliations:** ^1^ Predictive Society and Data Analytics Lab, Faculty of Information Technology and Communication Sciences, Tampere University, Tampere, Finland; ^2^ Institute of Biosciences and Medical Technology, Tampere, Finland; ^3^ Department of Computer Science, Swiss Distance University of Applied Sciences, Brig, Switzerland; ^4^ School of Science, Xian Technological University, Xian, China; ^5^ College of Artificial Intelligence, Nankai University, Tianjin, China; ^6^ Department of Biomedical Computer Science and Mechatronics, The Health and Life Science University, UMIT, Hall in Tyrol, Austria

**Keywords:** computational social science, data science, social data, web experiments, network science, prediction models, causal models, social sciences

## Abstract

The ultimate goal of the social sciences is to find a general social theory encompassing all aspects of social and collective phenomena. The traditional approach to this is very stringent by trying to find causal explanations and models. However, this approach has been recently criticized for preventing progress due to *neglecting prediction abilities* of models that support more problem-oriented approaches. The latter models would be enabled by the surge of big Web-data currently available. Interestingly, this problem cannot be overcome with methods from *computational social science* (CSS) alone because this field is dominated by simulation-based approaches and descriptive models. In this article, we address this issue and argue that the combination of big social data with social networks is needed for creating prediction models. We will argue that this alliance has the potential for gradually establishing a causal social theory. In order to emphasize the importance of integrating big social data with social networks, we call this approach *data-driven computational social network science* (DD-CSNS).

## 1 Introduction

The social sciences study the general behavior of groups, communities, and societies, and the interactions among such entities and their changes over time. This spans a wide number of questions from anthropology, sociology, economy, psychology, cyberpsychology, and political science (Kosinski et al., 2013; Badjatiya et al., 2017; Wei et al., 2017; Bail et al., 2018). Modern approaches to such interdisciplinary problems utilize computational methods and for this reason they have been called *computational social science* (CSS) (Lazer et al., 2009). An important aspect of methods from CSS are simulation-based approaches, e.g., agent-based modeling (Cioffi-Revilla, 2010; Conte et al., 2012; Conte and Paolucci, 2014; Holme and Liljeros, 2015). However, recent progress in information technology created new means to exchange digital information via social media, text messaging, or phone calls which led to a surge of data capturing a wealth of information about the underlying social behavior of individuals and groups. This opened new possibility and challenges at the same time because the resulting big social data cannot be analyzed in a simulation-based manner as, e.g., provided by CSS.

In this article, we move beyond CSS by presenting a new approach we call *data-driven computational social network science* (DD-CSNS). This new approach combines big social data with social networks for creating prediction models. Overall, this renders DD-CSNS as a data science because it integrates methods from network science and machine learning (Barabási, 2013; Conroy et al., 2015; Emmert-Streib and Dehmer, 2019). As such, it provides prediction models that can be practically utilized in a solution-oriented manner.

We will argue that the combination of big social data with network-based methods is the key for providing a pragmatic way forward to establish an explanatory model as social theory. In our opinion, so far, this combination has been largely overlooked and discussions focused either on opportunities provided by novel data (Hofman et al., 2017) or social networks (Wasserman and Faust, 1994), but neither the combination nor the use of networks as prediction models for the social sciences has been addressed adequately.

In contrast, our discussion of DD-CSNS is different with respect to the following points. First, we emphasize the integration of data-driven and network-based approaches for DD-CSNS. On one hand, this directly utilizes social data (González-Bailón, 2013; Chang et al., 2014; Shah et al., 2015) and on the other hand, it leverages the power of network models. Second, we are assuming large amounts of data. Nowadays, such data are called “*big social data*” (Olshannikova et al., 2017). In the social sciences, data with such characteristics are often Web-enabled, e.g., from social media or e-commerce platforms. Third, we will argue that the usage of networks has a dual meaning because it can either lead to predictive models or inferential models (also called causal models). We will discuss differences between both types of models from a statistical view and draw also parallels to systems biology because this field embraces already data-driven and network-based approaches, e.g., for studying genomics data. Importantly, the above points are not independent from each other but are interrelated. This makes the discussion intricate requiring also contextual information for appreciating the arguments put forward.

Overall, our article provides arguments that the combination of big social data with network-based approaches provides a pragmatic and efficient way forward toward establishing a causal social theory. Hence, our approach is complementary to a simulation-based view, see, e.g., Conte et al. (2012). We want to emphasize that by arguing in favor of DD-CSNS, we do not imply that this renders simulation-based studies as mute or inferior but that in the light of the current big social data surge a *data-driven computational social network science* (DD-CSNS) provides complementary qualities that deserve special attention (Chang et al., 2014).

## 2 Origin of the Problem

It has been widely acknowledged that the social sciences are facing a fundamental problem. Specifically, in Hofman et al. (2017) it has been pointed out that, traditionally, social scientists tried to find causal explanations of models describing human and social phenomena while neglecting predictive abilities and accuracies of such models. Unfortunately, this view has a long tradition going back to the 1960s making it nontrivial to address (Weber, 1968; Watts, 2014). As a solution, in Watts (2017) it has been argued that the social sciences should pursue a more solution-oriented approach. Despite the recognition of this problem, no practical solutions have been offered.

It is important to note the even modern approaches to the social sciences, for example, *computational social science* (CSS) (Lazer et al., 2009) do not provide dedicated solutions for this problem. The reason for this is that CSS is mainly based on simulations (dynamical systems, cellular automata, and agent-based modeling), social networks, social complexity (considering society as complex adaptive system), and big social data (Cioffi-Revilla, 2017) utilizing the individual computational approaches in a classical way. That means the main pillars of CSS are simulations and descriptions but not prediction models. Hence, none of such approaches provide solutions to the problems raised by Watts (2017).

In this study, we move beyond CSS in the following way. We argue that the combination of big social data with social networks enables the definition of prediction models which can be then utilized for solution-oriented approaches. Due to the fact that the combination of a data-driven and network-based approach is largely new to the social sciences this new ground needs to be conquered (Watts, 2016). In the reminder of the work, we will show that this approach can be practically implemented by current available means and data. Overall, in our opinion, this will provide a pragmatic way forward for gradually establishing a causal social theory via what we call in this study *data-driven computational social network science* (DD-CSNS).

**FIGURE 1:**
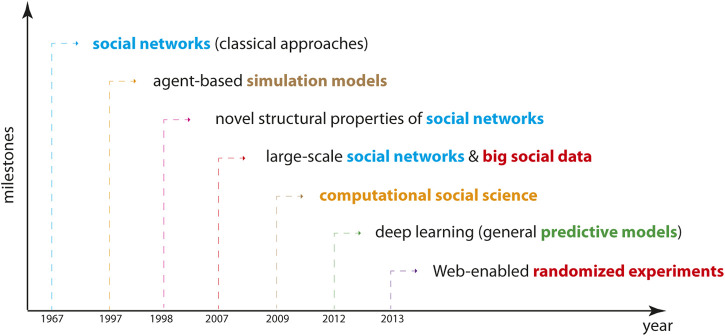
A time line of milestones in the social sciences. The shown years mark notable events of seminal contributions which all contribute to the definition of DD-CSNS. The specific milestones from left to right are studies by Milgram (1967), Axelrod (1997), Watts and Strogatz (1998), Mislove et al. (2007), Lazer et al. (2009), Krizhevsky et al. (2012), Muchnik et al. (2013).

In order to discuss our perspective on DD-CSNS, we organize the research according to the introduction of seminal contributions in the social sciences. A time line of these is shown in Figure 1. This provides a natural progression of components needed for establishing DD-CSNS. Here it is worth emphasizing that the contributions shown in Figure 1 are spanning several decades, whereas the first dates back to 1967 (Milgram, 1967). That means this is rather a slow progress and clearly many other contributions have been made during this time in the social sciences. However, none of those contributions is as important as the ones listed in Figure 1 for the establishment of DD-CSNS.

## 3 Importance of Networks for Social Sciences

The first component of DD-CSNS is provided by social networks. Historically, one needs to distinguish between three phases representing different types of social networks (Borgatti et al., 2009), namely 1) classical social networks, 2) novel structural properties of social networks, and 3) large-scale social networks.

The first phase started with a graphical representation of interactions among individuals utilized by Moreno, who is widely credited as one of the founders of social networks (Moreno, 1934), and studies using matrix algebra to investigate social circles and groups by means of networks (Luce and Perry, 1949). A milestone of this era is the study by Milgram investigating the average path length in social networks (Milgram, 1967), later called *six degrees of separation* (Kleinfeld, 2002). A similar influential contribution is from Granovetter who studied the spread of information in social networks (Granovetter, 1973). He found that weak ties in networks are especially crucial for enabling a far reaching spread of information, e.g., in marketing and politics. These studies informed the second phase of social networks where novel structural properties have been studied (Wasserman and Galaskiewicz, 1994). A milestone from this era is a research by Watts and Strogats introducing a mathematical network model with so-called *small-world* properties (Watts and Strogatz, 1998).

Despite the fact that social networks have been studied since the 1930s (see above), the structure of truly large-scale networks has been out-off reach until the mid 2000s, marking the third phase of social networks; see Figure 1. For instance, in an impressive study by Mislove et al. (2007) explains that over 10 million users of Flickr, YouTube, LiveJournal, and Orkut have been used to construct their underlying social networks together with more than 328 million links. This marked the beginning of a new era that is characterized by utilizing big social data for the construction and structural analysis of many other large-scale social networks (Leskovec and Horvitz, 2008; Manikonda et al., 2014; Myers et al., 2014). The structure of such networks has been studied in many ways, including community or motif detection, degree distributions, social circles, centrality indices or their structural evolution (Easley and Kleinberg, 2010; Kumar et al., 2010; Opsahl et al., 2010; Szell et al., 2010; Newman, 2012; McAuley and Leskovec, 2014). Since then many types of social networks have been studied in a data-driven way, for instance, in economy (Hidalgo and Hausmann, 2009; Emmert-Streib et al., 2018a) and finance (Mantegna, 1999; Baltakys et al., 2018). For an overview of many different large-scale social networks see the *Stanford Large Network Dataset Collection* (Leskovec and Krevl, 2014).

It is important to highlight that the third phase was only feasible due to the availability of big social data. For this reason, we provide in the next section a closer look at big social data and social media.

## 4 Big Social Data

**FIGURE 2:**
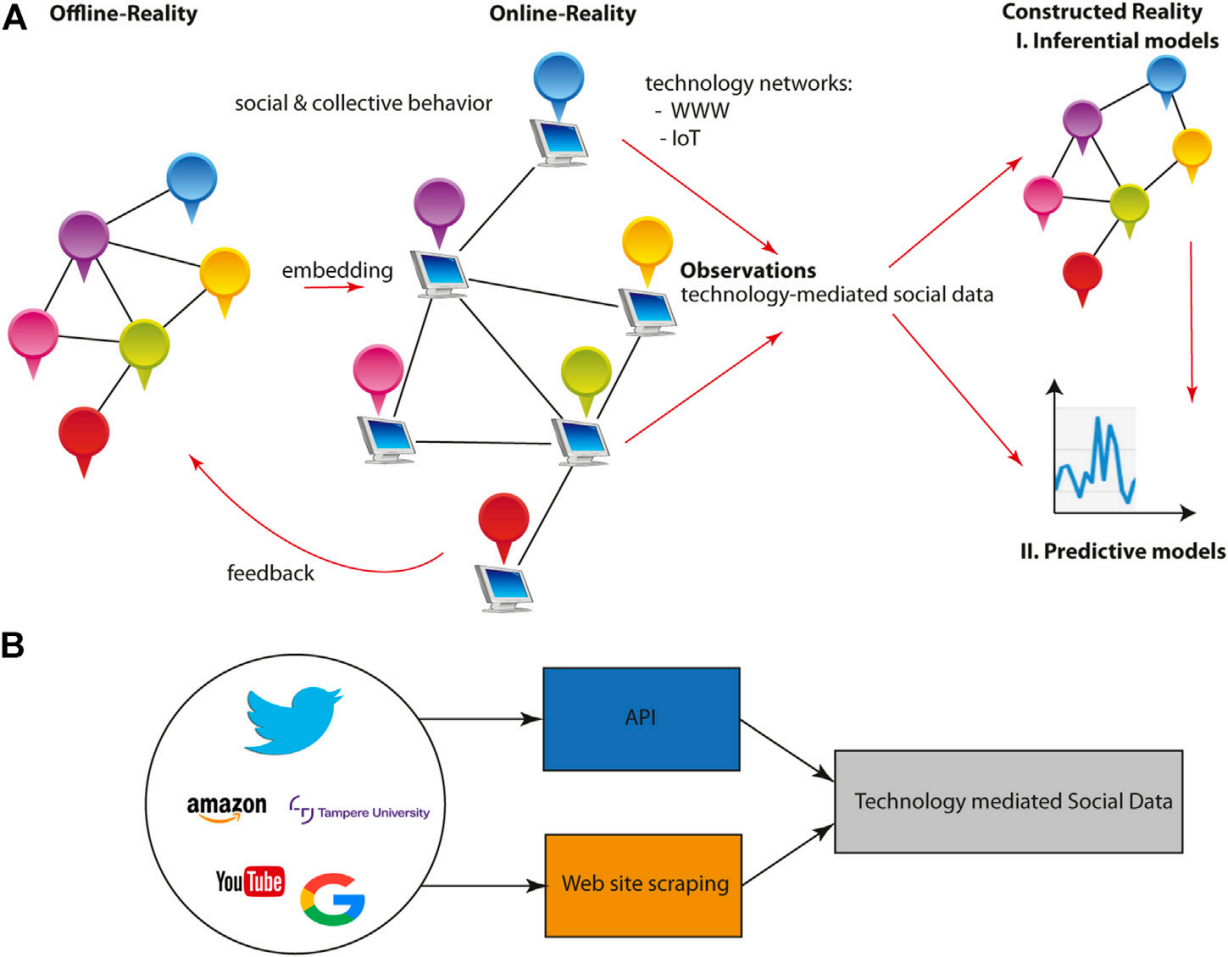
**(A)** Connection between offline-reality, online-reality, and constructed-reality. In online communication, individuals do not directly interact with each other but the communication is technology-mediated via computers, laptops, or phones. The resulting technology-mediated social data can then be used to create two different types of models: 1) inferential models also called explanatory models or 2) predictive models. Due to the fact that behind offline as well as online communication is a social network, the technology-medicated social data carry a network signature. **(B)** Converting Web information to social data requires usually an indirect approach either via an API (application programming interface) or Web site scraping. Only in exceptional cases, it will be possible to directly download the data.

The second component of DD-CSNS is provided by big social data. Due to the technology-mediated nature of big social data, e.g., via Web-enabled data (see Figure 2B), the collection of such data is governed by communication technologies, internet-based services, and sensor networks. Popular examples for such platforms are blogs (Blogger and Tumblr), social media (Facebook, Twitter, and YouTube), emails, cell phones, e-commerce (Amazon, iTunes, and eBay), online games, or social news sites (Reddit and Fark).

Differences between these social data generating instances are also reflected in the way the data are obtained. Specifically, we can distinguish three major ways for accessing data:

1. Downloading data from repositories
2. Accessing via API (application programming interface)
3. Web scraping

An example for the first type of data accessing is email data. Accessing via an API is only possible if a Web service offers such functionality. Examples for such Web services are Twitter, Facebook, or Amazon. Importantly, the first two data accessing types are either available or not, hence, this cannot be influenced by the users themselves. In contrast, Web scraping can always be used to gather data from a Web site. A disadvantage is that this requires proficiency in programming. Overall, most data can be accessed via method 2) and 3), whereas 2) should be always preferred if available.

Despite certain differences among the above platforms, all are fundamentally different from classical social science data generated, e.g., via surveys or interviews because neither has an interactive component. Hence, such technology-mediated social data provide a novel type of information to interrogate social phenomena. Furthermore, such data are also different in another aspect because they are “big.” This is also different to most survey-based social science data which have a very limited scope.

A final difference is that not only observational data can be gathered but randomized experiments can be conducted. For instance, in the study by Muchnik et al. (2013), the influence of social contacts has been studied on decision making. The authors analyzed whether the comments and ratings on a social news Web site affect the rating behavior of individuals. The importance of this study is that a randomized experiment has been designed by partnering with a social news website to conduct the experiment (Taylor and Eckles, 2018). One of their results showed that prior ratings led to a significant bias in the individual rating behavior, and positive and negative social influences led to an asymmetric herding effect. Such investigations are examples for virtual labs that utilize randomized controlled Web-based experiments for conducting a study (Kohavi et al., 2009).

Overall, these three characteristics make Web-enabled social data more potent compared to traditional data sources and allow the creation of high-quality social networks.

## 5 Network-Based Prediction Models

Finally, the third component of DD-CSNS is provided by prediction models. Importantly, this last component is not independent of the first two ones but builds upon these.

A visualization of this connection is shown in Figure 2A. Regardless of whether one is studying in groups, communities, or markets behind all such systems are social networks describing the interactions among those entities establishing their social and collective behavior. Hence, all social phenomena are inherently network-mediated. For online phenomena studying, e.g., communication via the WWW, there is a technology layer that enables the communication between individuals. That means each individual is connected to a computer or a phone, which is connected to another computer which is then connected to another individual. This provides a technology embedding of the underlying offline social network. Hence, the resulting observable social data generated from such interactions are technology-mediated, see Figure 2A. It is important to highlight that it has been pointed out that this gives not only information about the user behavior online but potentially about the general human social behavior (Strohmaier and Wagner, 2014). However, so far it is not entirely clear if this is unconditionally true or if this holds only in certain situations (Tang et al., 2014).

From such social data, two different types of prediction models can be recovered: 1) inferential models also called explanatory models and 2) predictive models. In order to obtain explanatory models, a reconstruction of the social networks is needed because without them no causal explanations can be given. However, even if one does not aim for the reconstruction of social networks, the social data themselves include the signature of the underlying (offline and online) social networks. Hence, regardless of what type of analysis one is aiming for, each such analysis is carrying information about the underlying social networks.

In order to demonstrate that the difference between an inferential and a predictive model is of fundamental nature, we discuss this issue in more detail.

### 5.1 Duality of Prediction and Inference

In the statistics literature, one distinguishes between two main types of models. The first type, called inferential or explanatory model, provides a causal explanation of the data generation process whereas the second type, called predictive model, just produces forecasts (Breiman, 2001; Shmueli et al., 2010). Ultimately, an inferential model is more informative than a predictive model because an explanatory model can make predictions but the predictive model does not provide (causal) explanations for such predictions. A prime example for an explanatory model is a causal Bayesian network or agent-based simulations, in contrast, a support-vector machine or a deep neural network are examples for prediction models.

Due to the complementary capabilities of predictive and inferential models they are coexisting and each is useful in its own right. Regarding a theory of social science, it would be desirable to be an explanatory model. However, until such a theory is feasible predictive models should be used to utilize the big social data to test and identify one hypothesis after another as building blocks for such a theory. This gives a pragmatic working direction for the way to go forward without abandoning the goal to aspire for a causal model as grant theory.

It is interesting to note that in systems biology predictive models and inferential models are coexisting since many years. For instance, differentially expressed genes are commonly identified using predictive models (Reiner et al., 2003) whereas for the inference of causal gene regulatory networks inferential models are used (Altay and Emmert-Streib, 2010).

A general problem for creating causal models from data is that their inference from observational data is very challenging requiring usually in addition also experimental data (for instance generated by perturbations of the system). Currently, most social data are observational data obtained from merely observing the social behavior and interactions among individuals. However, as discussed in Section 4, randomized Web experiments can be conducted for social media, at least under certain conditions.

## 6 DD-CSNS for Theory Discovery

**FIGURE 3:**
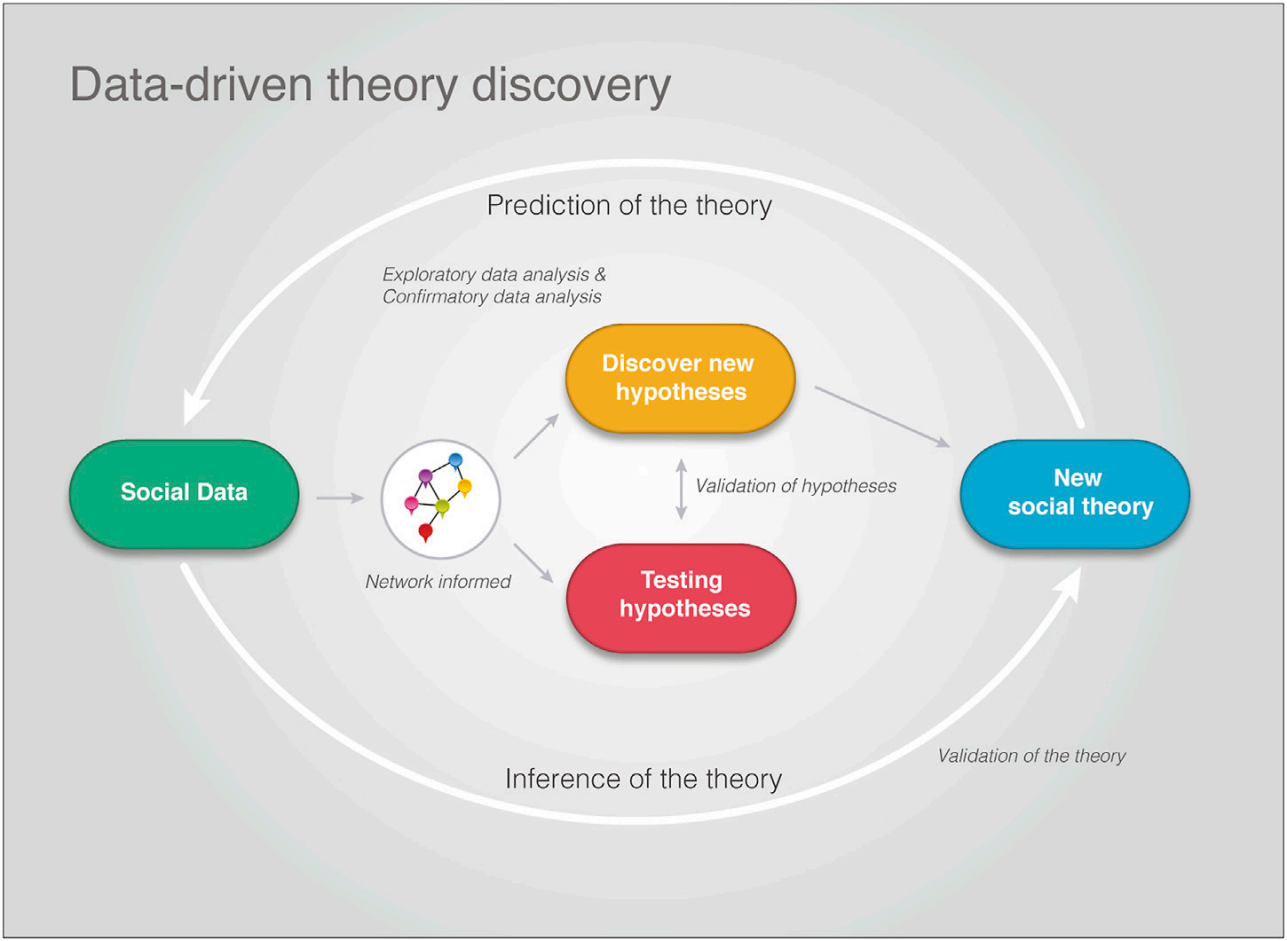
An overview of DD-CSNS for social theory discovery. Regardless of the type of model that is used (inferential or predictive), such models are network-informed capturing social interactions from the underlying phenomena under investigation. Importantly, the process of scientific discovery is a cyclically sequence of exploration, prediction and validation.

Finally, we can summarize our discussion concisely in a diagrammatic way as shown in Figure 3. In DD-CSNS, all work starts from social data gathered in various ways, see also the discussion in Section 4. Then, the social data, which are network-mediated, are used for an exploratory or confirmatory analysis to either discover or test hypotheses (see Section 5.1). We would like to re-emphasize that regardless of the type of model, such an analysis is always network-informed by social networks. Specifically, this could be done directly, e.g., via constructing social networks and exploring their structural meanings (Manikonda et al., 2014; Myers et al., 2014) or indirectly as in the study by Curme et al. (2014). Progressively, this will allow to build-up a social theory of collective phenomena. Importantly, even when such a social theory is only partially established, the existing knowledge can be utilized for making novel predictions about the underlying social phenomena that can be tested experimentally.

Overall, DD-CSNS describes an iterative process that forms a cyclical sequence of exploration, prediction and validation. We would like to highlight that this is also the generally accepted view on scientific discovery (Godfrey-Smith, 2003), regardless of the leap of progress. Interestingly, we recovered this process naturally by composition of the three individual components of DD-CSNS, i.e., social networks, social data and prediction models.

As a proof of concept for the above approach to DD-CSNS, we discuss in the following some case studies.

### 6.1 Case Studies: Social Contagion, Psychological Targeting, and Fake News

In sociology, it is well known that emotions can be transferred among people via emotional contagion (Fowler and Christakis, 2008). However, it is unclear if this can also occurs when people communicate only indirectly with each other via the Web. In Kramer et al. (2014), emotional contagion via news feed has been confirmed utilizing almost 1 million users with a Facebook account. Furthermore, in Hodas and Lerman (2014) conditions for social contagion have been established. The authors found that the spread of information via an individual is proportional to exposure frequency and positive feedback from friends. Both factors increase the likelihood of a response. Hence, the position within a social network strongly affects social contagion.

It is important to note that social contagion can be practically utilized for various applications. An example of this is provided by *psychological targeting* (Matz et al., 2017). Psychological targeting predicts the personality of users, e.g., via Facbook “Likes” (Youyou et al., 2015) and utilizes this information to influence the behavior of people by psychological mass persuasion. In Matz et al. (2017), it has been shown that psychological targeting can be used to effect the purchasing behavior of users, and hence, provides an efficient means to influence decision making.

In recent years, the topic of fake news detection received much attention. This was also triggered by the United States Presidential Election in 2016. In the study by Conroy et al. (2015) a categorization of detection methods was presented either utilizing linguistic cues (in combination with machine learning approaches) or network analyses. Furthermore, they proposed guidelines for fake news detecting methods (Conroy et al., 2015). As a result they found that automatic detection methods can lead to very good classification results; however, the results are very domain-specific.

At this point it seems necessary to add some words of caution. Specifically, studies about social contagion have been criticized on ethical grounds, e.g., if social media users of Facebook were informed sufficiently regarding the conducted experiments (Jouhki et al., 2016). As a consequence, currently, Facebook and others have stalled further experiments. However, if this decision is long lasting or if there are exceptions to this policy is unclear. Also, there are constantly new social media sites that could allow similar studies subject to their own regulations.

Overall, the above studies are examples for a combined usage of big social data for constructing social networks which are then utilized for making predictions about the underlying phenomenon. Interestingly, none of these studies casts the problem explicitly as a DD-CSNS framework by emphasizing the integration of the three components social networks, social data, and prediction models. Instead, this integration is done as a matter of course.

### 6.2 Practical Approach

In order to show how our iterative approach can be implemented practically, in the following we outline such a framework. It is very natural to start with the generation or collection of social data because this is usually the starting point of any investigation. Based on these social data one needs to decide if the social networks are i) directly given, e.g., from previous studies, ii) directly inferable based on the data, or iii) indirectly given. The latter point means that there is an underlying social network but the data may not be enough to infer such a network with sufficient quality neither may such a network be available from previous studies. As a result from this assessment, either a prediction model based on social networks or a network-independent prediction model is chosen for further analysis. Usually, this analysis step leads to novel insights about the underlying social phenomena, and hence, to new hypotheses. These insights can then be used to conduct new experiments which lead to new data giving rise to a new discovery circle; see Figure 3.

## 7 Discussion

In an inspirational article about *emergence* from 1972, Anderson argued that despite the fact that higher organized sciences, e.g., sociology or psychology, obey the laws of the previous hierarchy levels, these laws do not fully explain all observable phenomena (Anderson, 1972). For this reason his article has been titled “More is different,” whereas the difference accounts for the emergence of new behavior and phenomena. Hence, the laws of physics are not sufficient to explain our social behavior.

So what can be learned from sciences at lower hierarchy levels below the social sciences? Maybe the biggest leap of progress within the last few decades has been achieved in biology. Initially, it was purely gene-focused studies to explain phenotypes and disorders (Beadle and Tatum, 1941), and then the field shifted toward systems biology by embracing groundbreaking work by von Bertalanffy, Waddington, and Kaufman (von Bertalanffy, 1950; Waddington, 1957; Kauffman, 1969). Here, it is interesting to note that these studies date back to the 1950s and 1960s. Nowadays, it is well established that a functional understanding of biological, biomedical, and pharmacological problems can only be achieved via studying gene regulatory networks and their interactions inferred from big genomic data (Barabási, 2007; Vidal, 2009; Emmert-Streib et al., 2014; Emmert-Streib and Dehmer, 2018; Musa et al., 2019; Manjang et al., 2020). From a practical point of view, the human genome project paved the way for modern-high through technologies, especially for next-generation sequencing (Quackenbush, 2011). Unfortunately, despite this progress the grant theory for all those problems is still absent.

As a consequence from all this, one can draw the following lessons from biology that are relevant for the social sciences. First, due to the higher complexity level of the social sciences, that is, than that of biology, and the lack of a grant theory even for biology; it is not surprising that we are also lacking such a theory for the social sciences. Hence, from a pragmatic point of view, and given the availability of big social data, a data-driven approach—as in biology—seems currently the best step forward to advance our knowledge and understanding of social phenomena and to build-up a theory in a gradual manner. Second, in biology the go-to method for dealing with big genomic data is the study of networks (Barabási and Oltvai, 2004). Given the fact that biological as well as social systems are multiscale, complex, and having an emergent nature, it is no surprise that *networks* are also at the heart of many social science studies in the form of social networks (Milgram, 1967; Freeman, 1979; Wasserman and Faust, 1994; Borgatti et al., 2009). Hence, the study of social networks should be further advanced and utilized, for instance, for prediction making. Predictions can be naturally obtained from predictive models (see Figure 2) but also from inferential models, and by empirically testing such predictions a social theory can grow gradually. In addition, this provides a direct answer to the solution-oriented approach suggested by Watts (2017).

Interestingly, such an approach could also provide a natural interface to current work in machine learning about deep learning networks in a two-fold way (LeCun et al., 2015). First, deep learning-based methods have been shown to result in superior predictive power compared to standard machine learning and statistics models (Lee et al., 2009; Cireşan et al., 2012; Emmert-Streib et al., 2020a). Hence, such methods could also be of great usage for social problems aiming to make accurate predictions about social or behavioral phenomena (Emmert-Streib et al., 2018b). Second, deep learning networks are frequently criticized for lacking interpretability and explainability (Lipton, 2016; Xu et al., 2019; Emmert-Streib et al., 2020b). Interestingly, this lack might be overcome by utilizing social networks underlying big social data for informing the deep network architectures. This could potentially lead to an interpretable network structure and at the same time provide high-quality predictions.

In a widely noted article by Borgatti et al. (2009) the authors wrote (second sentence in abstract): “For social scientists, the theory of networks has been a gold mine, yielding explanations for social phenomena in a wide variety of disciplines from psychology to economics.” Considering that this could be achieved without fully exploiting network-based prediction models and the big social data provided by social media that have been emerged only during the last decade, the potential of a *data-driven computational social network science* (DD-CSNS) can hardly be overestimated.

Finally, we would like to add that there is another interesting connection between Borgatti et al. (2009) and Anderson (1972) (see above) that is worth discussing. Specifically, Borgattie et al. compared the social sciences with physics, whereas Anderson emphasized that on the complexity ladder, biology is situated between physics and sociology making the comparison between biology and the social sciences more fair. This should become especially clear considering that there are generally accepted physical theories of general relativity (Wald, 2010) and quantum mechanics (Griffiths and Schroeter, 2018), yet the combination of both is still an outstanding problem (Rovelli, 2004). In contrast, there is unarguable no comparable, even 312 partial, theory for the social sciences (Remark: By *theory* we mean a mathematical, formal model that allows to make quantitative, testable predictions about observable phenomena, hence, even contributions like the theory of society by Luhmann (2012) do not provide such a theory in the strict sense). For this reason, we added above a brief outline of the development of biology from its gene-centered beginnings toward its current data-driven state where network-based methods serve as prediction models. Hence, in our opinion, biology is the role model for the social sciences, including psychology and economics that can give a glimpse of what lies ahead of us and what can be realistically expected within the foreseeable future. For reasons of completeness, we would like to note that the concept of emergence has been controversially discussed in the social sciences (Elder-Vass, 2007).

In summary, our discussion above introduced DD-CSNS gradually because this reflects also the natural progression of the social sciences including seminal contributions thereof over time, as outlined in Figure 1. In this way, we wanted to highlight that there is not just one idea on which DD-CSNS is based on but there are in fact three key components that all contribute collectively in a mutually informing way, namely, social networks, social data, and prediction models. For reasons of clarity, we would like to mention that a major concern of our contribution is the clear explication of these concepts and their importance rather than in claiming that so far no study applied these principles in some form.

## 8 Conclusion

In this article, we outlined a pragmatic way forward for establishing a causal social theory based on a *data-driven computational social network science* (DD-CSNS). Frequently, social phenomena are discussed in the context of physical models as a desirable form of causal models. We think that despite the beauty and success of physical models, also outside of physics—for instance in chemistry—physics is too far down the complexity ladder to make a fair role model for the social sciences, especially, considering previous criticisms (Watts, 2014; Hofman et al., 2017). Instead, in this article, we used the network-based models in systems biology as a role model for a comparison and as guideline. Such models might give a more realistic view on how a possible future theory of the social sciences might look.

In a much-noticed article by Anderson (2008), it has been somewhat gloomily argued that “the data deluge makes the scientific method obsolete.” In contrast, in this study, we brought forward the view of DD-CSNS as a data science. DD-CSNS utilizes data, yet at the same time, it puts the science back into “social” via network-based prediction models.
